# Emerging Technologies for the Detection of Rabies Virus: Challenges and Hopes in the 21st Century

**DOI:** 10.1371/journal.pntd.0000530

**Published:** 2009-09-29

**Authors:** Anthony R. Fooks, Nicholas Johnson, Conrad M. Freuling, Philip R. Wakeley, Ashley C. Banyard, Lorraine M. McElhinney, Denise A. Marston, Akbar Dastjerdi, Edward Wright, Robin A. Weiss, Thomas Müller

**Affiliations:** 1 Rabies and Wildlife Zoonoses Group, Veterinary Laboratories Agency (VLA, Weybridge), WHO Collaborating Centre for the Characterisation of Rabies and Rabies-related Viruses, New Haw, Addlestone, United Kingdom; 2 Friedrich-Loeffler-Institute, Federal Research Institute of Animal Health, Wusterhausen, Germany; 3 Division of Infection and Immunity, University College London, London, United Kingdom; Centers for Disease Control and Prevention, United States of America

## Abstract

The diagnosis of rabies is routinely based on clinical and epidemiological information, especially when exposures are reported in rabies-endemic countries. Diagnostic tests using conventional assays that appear to be negative, even when undertaken late in the disease and despite the clinical diagnosis, have a tendency, at times, to be unreliable. These tests are rarely optimal and entirely dependent on the nature and quality of the sample supplied. In the course of the past three decades, the application of molecular biology has aided in the development of tests that result in a more rapid detection of rabies virus. These tests enable viral strain identification from clinical specimens. Currently, there are a number of molecular tests that can be used to complement conventional tests in rabies diagnosis. Indeed the challenges in the 21st century for the development of rabies diagnostics are not of a technical nature; these tests are available now. The challenges in the 21st century for diagnostic test developers are two-fold: firstly, to achieve internationally accepted validation of a test that will then lead to its acceptance by organisations globally. Secondly, the areas of the world where such tests are needed are mainly in developing regions where financial and logistical barriers prevent their implementation. Although developing countries with a poor healthcare infrastructure recognise that molecular-based diagnostic assays will be unaffordable for routine use, the cost/benefit ratio should still be measured. Adoption of rapid and affordable rabies diagnostic tests for use in developing countries highlights the importance of sharing and transferring technology through laboratory twinning between the developed and the developing countries. Importantly for developing countries, the benefit of molecular methods as tools is the capability for a differential diagnosis of human diseases that present with similar clinical symptoms. Antemortem testing for human rabies is now possible using molecular techniques. These barriers are not insurmountable and it is our expectation that if such tests are accepted and implemented where they are most needed, they will provide substantial improvements for rabies diagnosis and surveillance. The advent of molecular biology and new technological initiatives that combine advances in biology with other disciplines will support the development of techniques capable of high throughput testing with a low turnaround time for rabies diagnosis.

Validated diagnostic tests that confirm the presence of rabies virus or a lyssavirus variant have been the foundation of rabies control strategies in many countries. Historically, histopathological techniques such as the Sellers Stain technique [1] were used to determine the presence of Negri bodies as rabies virus-specific antigen, however due to poor sensitivity and specificity this technique is no longer recommended by the World Health organization (WHO). The Fluorescent Antibody test (FAT) [2] relies on the ability of a detector molecule (usually fluorescein isothiocyanate) coupled with a rabies specific antibody forming a conjugate to bind to and allow the visualisation of rabies antigen using fluorescent microscopy techniques. Microscopic analysis of samples is the only direct method that allows for the identification of rabies virus-specific antigen in a short time and at a reduced cost, irrespective of geographical origin and status of the host. It has to be regarded as the first step in diagnostic procedures for all laboratories. Autolysed samples can, however, reduce the sensitivity and specificity of the FAT. The Rabies Tissue Culture Infection Test (RTCIT) [3] and the Mouse Inoculation Test (MIT) [4] are based on the propagation and isolation of the virus. These diagnostic tests are used to detect virus particles either directly in tissue samples (FAT) or indirectly in animals and in tissue culture (MIT and RTCIT, respectively). The rationale for the use of virus isolation (RTCIT/MIT) from a sample where there is a suspicion of infection with rabies virus is always recommended, especially when Koch's postulates are likely to be met. Such amplification of the viral pathogen facilitates additional molecular analysis to be undertaken, including sequencing of the viral isolate and subsequent phylogenetic analysis. Conventional diagnostic tests for rabies (FAT, RTCIT, MIT) are not labour intensive and rely upon low throughput. The FAT can be completed in less than two hours. In contrast, both the RTCIT and MIT require longer turnaround times (4-days and 28-days, respectively).

The fluorescent antibody virus neutralisation (FAVN) test [5] and the Rapid Fluorescent Focus Inhibition Test (RFFIT) [6] utilise a similar principle, to measure the level of virus neutralising antibody in vaccinated individuals. ‘Indirect’ serological methods, including the FAVN and RFFIT measure the host response to infection/vaccination only and do not detect the presence of infectious virus/antigen directly. However, host antibody detection (FAVN/RFFIT) is an indirect tool to measure the presence of rabies virus in a non-immunised individual by evaluating the host response to infection. The test may lack sensitivity and specificity, and the interpretation of the test results may be difficult as the host response to infection varies substantially between individuals. As such, the negative predictive value of serological tests for rabies diagnosis is considered poor. Therefore, serological assays are not suitable as diagnostic tools for routine rabies testing.

These internationally approved methods have provided accurate and timely information of animal and human rabies cases thereby supporting surveillance for rabies and providing a greater understanding of the epidemiology of this disease (Box 1). For numerous laboratories in rabies-endemic regions in the developing world, cost and simplicity are critical factors in the delivery of disease diagnosis and cannot be neglected, even when the principal consideration is for rapid diagnosis. Therefore, cost and simplicity need to be considered if new technologies are to be adopted in the regions of the world where they are most needed.

**Box 1. Key Learning Points**

1. Validated diagnostic tests capable of confirming the presence of rabies virus in clinical samples have improved the quality, accuracy and speed of rabies diagnosis in many national reference laboratories thereby supporting rabies control strategies with the global vision of dog rabies elimination in developing countries. Ante-mortem testing for human rabies is now possible using molecular techniques.
2. Adoption of rapid and affordable rabies diagnostic tests for use in developing countries highlights the importance of participation in projects that link laboratories from the developed and the developing countries.
3. The advent of molecular biology and new technological initiatives that combine advances in biology with other disciplines will support the development of microchip, biosensor and robotics-based techniques capable of high throughput testing with a low turnaround time for rabies diagnosis.

Molecular tools based on the detection and manipulation of the genetic information of the virus are becoming more widely accepted and accessible for the diagnosis of rabies. The advent of molecular biology is changing the face of diagnostic virology generally enabling high throughput and short turnaround-time analysis of samples. In the 21^st^ century, it is expected that diagnostic virology techniques for high throughput rabies virus detection will progress rapidly towards the use of molecular diagnostics replacing more conventional testing techniques such as virus isolation and histopathology. It is also possible that immunological tests, measuring ‘surrogate’ markers such as cytokines and electrolytes, will augment the standard diagnostic approach; nevertheless they will continue to remain oddities outside the realms of the routine diagnostic laboratory and be confined to a few reference laboratories. Semi-automated or automated instruments and robotics-based techniques are useful when large numbers of the same test are undertaken and these tests will continue to increase in popularity and use, especially in central reference laboratories rather than in each local or regional facility. New technological advances will undoubtedly be faster, more accurate and may, in time, offer a cost-effective alternative to traditional rabies diagnostic tests. These paradigm shifts including modern advances in technology will lead to the effective control of rabies in animals and wildlife [7] (Box 2). This review provides information on some of the latest developments and diagnostic techniques for determining the presence of rabies virus or nucleic acid in diagnostic samples.

**Box 2. Key Manuscripts in the Field**

1. Barrat J (1996) Simple technique for the collection and shipment of brain specimens for rabies diagnosis. In: Meslin FX, Kaplan MM, Koprowski H, editors. Laboratory techniques in rabies. Geneva: World Health Organisation. pp 425–432.
2. Bourhy H, Kissi B, Tordo N (1993) Molecular Diversity of the Lyssavirus Genus. Virology 194: 70–81.
3. Rupprecht CE, Hanlon CA, Slate D (2006) Control and prevention of rabies in animals: paradigm shifts. Developments in Biologicals 125: 103–111.
4. Sacramento D, Bourhy H, Tordo N (1991) PCR technique as an alternative method for diagnosis and molecular epidemiology of rabies virus. Molecular Cellular Probes 5: 229–240.
5. Willoughby RE Jr, Tieves KS, Hoffman GM, Ghanayem NS, Amlie-Lefond CM, Schwabe MJ, Chusid MJ, Rupprecht CE (2005). Survival after treatment of rabies with induction of coma. N. Engl. J. Med. 16: 2508–1514.

The principal focus of this review is to highlight the new developments in virology related to techniques for the diagnosis and surveillance of rabies. Literature reviews were identified through Web of Science, PubMed and Scopus using various search phrases. This review also drew on information provided to international organisations, mainly WHO and OIE, funded by the UK Department for Environment, Food and Rural Affairs (Defra) in an advisory context on diagnostic and surveillance strategies for rabies. This review however, does not reflect the views of Defra, WHO or OIE. This review provides information on some of the latest developments and diagnostic techniques for determining the presence of rabies virus in diagnostic samples. Our aim is to provide a viewpoint on the current thinking in diagnostic virology for rabies, reflecting the ‘neglected’ nature of this tropical disease and the contrasting needs of diagnostic laboratories in developed and developing countries.

## Antigen detection–based assays

### Development of Rapid Immunohistochemical Test (dRIT) in the evaluation of suspect rabies tissue samples

A direct Rapid Immunohistochemical Test (dRIT) for the postmortem detection of rabies virus antigen in brain smears has been developed [8]. Using a cocktail of highly concentrated and purified biotinylated monoclonal antibodies, rabies antigen can be detected by direct staining of fresh brain impressions within 1 hour. This test employs anti-rabies monoclonal antibodies specific for the nucleoprotein, a viral protein produced in abundance during productive infection. The FAT is based on antibodies specific for the same protein but, being conjugated to fluorescein isothiocyanate, requires a fluorescent microscope to visualise any specific antibody bound to viral protein within the test sample [2]. In contrast, the new dRIT antibody cocktail is biotinylated such that following a short incubation with a streptavidin-peroxidase complex, antibody-antigen binding complexes can be visualised through the addition of the substrate, 3-amino-9-ethylcarbazol. Performed on brain tissues, the dRIT has proven as sensitive as the FAT for fresh specimens [9],[10]. Brain impressions stained using the dRIT technique can be read within one hour and the antibody cocktail used has been shown to detect classical rabies virus strains (genotype 1) that have been assessed [11]. Currently, the FAT is routinely used to detect virus antigen in badly decomposed sample material. For the purpose of testing samples in the developing world where suitable cold storage for samples is often unavailable, this factor is important in the development of new tests. This obstacle has been overcome through evaluating sample preservation in phosphate buffered 50% glycerol at a range of temperatures for different time periods prior to testing for virus antigen. Glycerol saline solutions have been previously recognised as suitable storage media for tissue samples in the absence of cold storage [12] (Box 2). Using the dRIT in field studies in Tanzania, viral antigen could be detected in samples after considerable time periods post collection regardless of the regimen of glycerol preservative used [11]. Applications of the dRIT to analyse field samples in other rabies endemic regions have also proven highly successful. Field trials in Chad sought to study the dRIT in direct comparison to the FAT to attempt to confirm previous studies as to the incidence of rabies within a district known to be endemic. In this study, results between the two tests were 100% in agreement [9] and the only issue regarding use of the dRIT over the FAT was the need for the dRIT kit to be stored refrigerated prior to use. The dRIT will enable developing countries to perform routine rabies surveillance at greatly reduced cost and without the need for prohibitively expensive microscopic equipment along with the expertise and financial input needed to maintain them. The cost effectiveness of the dRIT will allow knowledge and technology transfer to areas of the developing world that currently are unable to diagnose rabies cases.

### Immunochromatographic techniques

Another method for the detection of rabies virus antigen from postmortem samples is a recently developed rapid immunodiagnostic test (RIDT) based on the principles of immunochromatography [13]. The immunochromatographic lateral flow strip test is a one-step test that facilitates low-cost, rapid identification of various analytes including viruses [14]. Briefly, suspect material is subjected to a test device similar to a lateral flow device. Conjugated detector antibodies attached to two different zones on a membrane indicate the presence of viral antigen. Preliminary validation studies with a limited number of samples showed that the RIDT might have great potential as a useful method for rabies diagnosis without the need for laboratory equipment [13]. However, thorough validation including various circulating variants of RABV and other lyssaviruses is still needed before this test could be relied upon and be used as an alternative for the gold standard FAT.

## Nucleic acid detection–based assays

### Reverse-transcriptase polymerase chain reaction (RT-PCR)

Various conventional RT-PCR protocols for the diagnostic amplification of lyssavirus genome fragments have been published (Tables 1–3). Since primers were selected from conserved regions of the genome, most assays amplify parts of the nucleoprotein (N-) gene as earlier proposed [15]. In generic approaches intended to detect all lyssaviruses either hemi-nested or fully nested amplifications are used and have applications for both antemortem (saliva, CSF, brain) and postmortem samples (principally brain tissue) (Table 2). Some of these diagnostic procedures are also applied for further virus characterization, including sequencing reactions [16] or restriction fragment length polymorphism (RFLP) [17]. Also, strain-specific RT-PCRs have been developed to distinguish various rabies virus (RABV) strains in a particular region [18].

**Table 1:** Conventional, gel-based PCR-assays for the detection rabies virus.

PCR	Primer name	Direction	Sequence	Details	Position	Fragment length	Author
heminested	20R	R	AGCTTGGCTGCATTCATGCC				[65]
	21F	F	ATGTAACACCCCTACAATG		55–73	210	
	23F	F	CAATATGAGTACAAGTACCCGGC			122	
nested	RabN1	F	GCTCTAGAACACCTCTACAATGGATGCCGACAA	1st round	59–84	1477	[18]
	RabN5	R	GGATTGAC(AG)AAGATCTTGCTCAT		1514–1536		
	RabNF	F	TTGT(AG)GA(TC)CAATATGAGTACAA	2nd round	135–156	762	
	RabNR	R	CCGGCTCAAACATTCTTCTTA		876–896		
heminested	P510	F	ATA GAG CAG ATTTTC GAG ACA GC		510-531		[66]
	P942	R	CCC ATA TAA CAT CCA ACA AAG TG	1st round	965-942	455	
	P784	R	CCT CAA AGT TCT TGT GGAAGA	2nd round	805-784	295	
standard	N 1161	F	AAG AAC TTC AAG AAT ACG AGG C		1161-1182	418	[67]
	N 1579	R	TTC AGC CAT CTC AAG ATC GG		1579–1560		
standard	113	F	GTAGGATGATATATGGG	RT only			[20]
	509	F	GAGAAAGAACTTCAAGA			377	
	304	R	GAGTCACTCGAATATGTC				
standard		F	ACT GAT GTA GAA GGGAAT TG	N-gene		533	[68]
		R	GAA CGG AAG TGG ATG AAA TA				
		F	TAA TCC CAG AGA TGC AAT	G-gene		406	
		R	CCT CAC AGT CTG GTC TCACC				
nested	Primer 1	F	GAAGCCTGAGATTATCGTGG	1st round	63–82	304	[23]
	Primer 2	R	CCCTTCTACATCAGTACG		349–367		
	Primer 3	F	TGAGTACAAGTACCCTGC	2nd round	90–107	139	
	Primer 4	R	GGAACATACATCGTCAGG		229-211		
standard	N1	F	TAGGGAGAAGGATCGTGGAGCACCATACTCTCA		611-632	179	[51]
	N2	R	GATGCAAGGTCGC ATATGAGTACCAG CCCTGAACAGTCTTCA		790-769		
standard	N12	F	GTAACACCTCTACAATGG		57-74		[52]
	N40	R	GCTTGATGATTGGAACTG		1368-1349		
nested	N1	F	TTT GAG ACT GCT CCT TTT				[22]
	N4	R	GCT TGA TGA TTG GAA CT				
	JW 4	F	AGAATGTTTGAGCCACGGCA				
	JW 5	R	TCAGGTGAAACCAGAAGTCC				

**Table 2 pntd-0000530-t002:** Conventional, gel-based PCR-assays for the generic detection of all lyssavirus species.

Genotype	PCR	Primer name	Direction	Sequence	Details	Position	Fragment length	Author
All	standard	N1	F	TTT GAG ACT GCT CCT TTT		587-605	443	[26]
		N2	R	CC CAT ATA GCA TCC TAC		1029-1013		
	heminested	JW12	F	ATGTAACACCYCTACAATG	universal lyssavirus primer	55–73		[69]
		JW6 (DPL)	R	CAATTCGCACACATTTTGTG	1st round (DUVV, RABV, LBV)	660–641	605	
		JW6 (E)	R	CAGTTGGCACACATCTTGTG	1st round (EBLV-1 and 2)			
		JW6 (M)	R	CAGTTAGCGCACATCTTATG	1st round (MOKV)			
		JW10 (DLE2)	R	GTCATCAAAGTGTGRTGCTC	2nd round (DUVV, LBV, EBLV-2)	636–617	581	
		JW10 (ME1)	R	GTCATCAATGTGTGRTGTTC	2nd round (MOKV, EBLV1)			
		JW10 (P)	R	GTCATTAGAGTATGGTGTTC	2nd round (RABV)			
	nested	D017	F	AGATCAATATGAGTAYAARTAYCC	2nd round forward primer instead of JW12, otherwise identical to Heaton et al.	139–163	497	[70]
	nested	LISEBL1F	F	AAGATGTGTGCCAACTGGAG	1st round			[71]
		LISEBL1R	R	ATGTTTGAGCCAGGGCAAGA				
		LISEBL2F	F	TACTGCTTATGAGGATTGTTC	2nd round			
		LISEBL2R	R	AAGAACTTCGAGGAAGAGATC				
	nested	GRAB1F	F	AARATNGTRGARCAYCACAC	1st round	538-557	373	[72]
		GRAB1R	R	GCRTTSGANGARTAAGGAGA		911-892		
		GRAB2F	F	AARATGTGYGCIAAYTGGAG	2nd round	574-593	259	
		GRAB2R	R	TCYTGHCCIGGCTCRAACAT		833-814		

**Table 3:** Conventional, gel-based PCR-assays for the detection of lyssavirus species other than RABV.

Genotype	PCR	Primer name	Direction	Sequence	Details	Position	Fragment length	Author
EBLV-1	hemi-nested	N1001fw		CAGAGTTGTGCACCCCATGAA		1061-1081	475	[73]
		1066fw		GAGAGAAGATTCTTCAGGGA		1136–1155	400	
		304rv		TTGACAAAGATCTTGCTCAT		1517-1536		
	nested	N1161	F	AAGAGCTACAGGATTACGAGG	First round	1161-1181	373	[74]
		N1534	R	GACAAAGATCTTGCTCATGA		1534-1514		
		EBLV-1nF	F	TTGGCAGATGATGGGACAGT	Second round	1211-1230	216	
		EBLV-1nR	R	TCCCTTATCTAATCAGGGGA		1427-1408		
EBLV-2	nested	EBLV-2F	F	TCATGGTCAATGGGGGAAAG	First round	1226–1245	229	[74]
		EBLV-2R	R	TTGGGATGGAGCAGGAAGAG		1455–1436		
		EBLV-2nF	F	CAAAAATCCCACATCAAAAG	Second round	1249–1269	180	
		EBLV-2nR	R	TCTTAGTTTTTTTCTTTCCCC		1429–1409		
LBV	standard	LagNF	F	GGGCAGATATGACGCGAGA				[75]
		LagNR	R	TTGACCGGGTTCAAACATC				
LBV, WCBV, MOKV	nested	N1F	F	ATGGAKTCWGAMAASATTGT	First round	71-90	595	[76]
		N550B	R	GTRCTCCARTTAGCRCACAT		647-666		
		N70F	F	GAYCAATATGARTATAARTA	Second round	140-159	439	
		N490B	R	TCCATYCTRTCTGCWACATT		560-579		
WCBV	nested		F	AATATCCTGCCATTACAGATTA	First round	165-177	606	[76]
			R	TCATATGCAGTGACAACTGTGC		750-771		
			F	ATGATGTGTGCTCCTATCTAGCA	Second round	273-295	394	
			R	GGTACTCCAATTGGCACACATC		646-667		
ABLV	hemi-nested	NP1087	F	GAGAAAGAG[A/C]T[G/T]CAAGA[A/C/T]TA		N-gene		[77]
		NP1279	R	CAG AGACATATCT[G/C]C[G/T][G/T]ATGTG				
		NP1227	R	CTTCA [C/T]C[G/T]ACC[A/T][C/T][C/T]GTTCATCAT)				
ARV	nested	F	F	AGTATCCTGCCATTGGGAATCA	First round	86–107	616	[28]
		R	R	GACTGTTGTAACTGCATATGAG		681–702		
		NF	F	ATGACGTCTGTTCTTATCTTGCT	Second round	203–225	376	
		NR	R	ATGTGTGCAAATTGGAGCACG		577–597		
KHUV	nested	F	F	AATACCCGGCTATTGTGGATAG	First round	86–107	615	[28]
		R	R	GAACTGTTGTGACCGCATATGA		680–701		
		NF	F	ATGATGTCTGTTCTTATTTGGCC	Second round	203–225	375	
		NR	R	AATGTGTGCTAACTGGAGCACA		576–597		
IRKV	nested	F	F	AATACCCTGCGATTGATGATAA	First round	86–107	615	[28]
		R	R	GAACAGTTGTGACTGCATATGA		680–701		
		NF	F	ATGATGTGTGCTCTTACTTGGCT	Second round	203–225	375	
		NR	R	AATGTGTGCAAATTGGAGCACA		576–597		

Classical RT-PCR assays proved to be a sensitive and specific tool for routine diagnostic purposes [19],[15], particularly in decomposed samples [20],[21],[22] or archival specimens [23],[24]. The sole detection of amplified RT-PCR products by gel-based systems however, especially when using hemi-nested RT-PCRs generates the risk of cross-contamination, does not allow an exact quantification of genome copies and does not include tests for specificity [25]. Hybridisation methods [26] and PCR-ELISA methods were established to overcome these difficulties [27], although these techniques have not become universally accepted. Additionally, many laboratories now use partial sequencing to confirm the detection of a lyssavirus and obtain data that can be used in a phylogenetic analysis of viruses circulating in a specific region. The importance of sequencing the PCR products was highlighted in an experimental study [28]. This study demonstrated that although the nested RT-PCR was shown to be the most sensitive of the diagnostic techniques employed, host genomic amplicons of the same size as the target amplicons were observed on the agarose gels, which were subsequently confirmed as false positives following direct sequencing [28].

### TaqMan RT-PCR

With the introduction of fluorogenic probes, detection of sequence specific templates can be achieved in real-time. Specificity is ensured by an inherent hybridization reaction, and cross-contamination is avoided due to the closed tube nature of the test [29],[30]. Consequently, for RABV and other lyssaviruses, various PCR assays using TaqMan technology have been described (Tables 4–5). A generic real-time TaqMan-PCR for the detection and differentiation of lyssavirus genotypes 1, 5, and 6 has also been developed [31]. This assay utilises a pan-lyssavirus primer set, which has been shown to amplify a large panel of representative lyssaviruses, with probes specifically designed to discriminate between classical rabies virus and the European Bat Lyssaviruses type-1 and -2 (EBLV-1 and EBLV-2). PCR assays using TaqMan technology have applications for antemortem and postmortem samples. The pan-lyssavirus primer set can also be used in conjunction with a specific dye such as SYBR Green to allow for rapid detection of the amplicons. Validation of probe based assays relies on the availability of representative viruses or nucleic acid. However, for some lyssavirus genotypes only a limited number of viruses or sequences are available for primer/probe design, and they may not represent the genetic diversity of all current variants that are circulating. Single mutations for the North American RABV strains [32] in the region of the primers or the probe can alter the sensitivity of the PCR. Thus the genetic diversity among lyssaviruses may hamper the use of a single assay for all lyssaviruses. As such scanning surveillance may benefit more from the use of a pan-lyssavirus primer SYBR green assay rather than a strain or specific based assay.

**Table 4:** Real-time PCR-assays for the detection of RABV.

PCR	Primer/Probe name	Role	Sequence	Position	Author
TaqMan	JW12	F	ATGTAACACCYCTACAATG	55–73	[31]
	N165–146	R	GCAGGGTAYTTRTACTCATA	165–146	
	LysGT1	P	ACAAGATTGTATTCAAAGTCAATAATCAG	81–109	
TaqMan	Probe	P	AAGCCCAGTATAACCTTAGGAAA	112 - 134	[65]
	23F	F	CAATATGAGTACAAGTACCCGGC		
	20R	R	AGCTTGGCTGCATTCATGCC		
SybrGreen(c)	O1	F	CTACAATGGATGCCGAC	66–82	[78]
	R6	R	CCTAGAGTTATACAGGGCT	201–183	
TaqMan	RB probe	P	TCAATTCTGATGACGAGGATTACTTCTCCGG		[51]
	1129F	F	CTGGCAGACGACGGAACC	1129	
	1218R	R	CATGATTCGAGTATAGACAGCC	1218	
TaqMan	gt1L	F	TACAATGGATGCCGACAAGA		[79]
	gt1P	R	CAAATC TTTGATGGCAGGGTA		
	AWgt1	P	TCAGGTGGTCTCTTTGAAGCCTGAGA		
TaqMan	AZ-EF	F	GAATCCTGATAGCACGGAGGG	278–298	[32]
		R	CTTCCACATCGGTGCGTTTT	333–352	
		P	CAAGATCACCCCAAATTCTCTTGTGGACA	303–331	
	AZ-SK	F	GTCGGCTGCTATATGGGTCAG	943–963	
		R	ATCTCATGCGGAGCACAGG	995–1013	
		P	TGAGGTCCTTGAATGCAACGGTAATAGCC	965–993	
	CASK	F	TCATGATGAATGGAGGTCGACTC	1226–1247	
		R	TTGATGATTGGAACTGACTGAGACA	1296–1272	
		P	AGAGATCGCATATACGGAGAT	1249–1270	
	NCSK	F	GGTGAAACCAGAAGTCCGGAA	1189–1209	
		R	CCGTATATGCGATCTCTTTAGTCGA	1266–1242	
		P	CTGTCTATACTCGAATCATGA	1211–1227	
	RAC	F	TGGTGAAACCAGGAGTCCAGA	1188–1208	
		R	ATCTTTTGAGTCGGCCCCC	1255–1235	
		P	CGGTCTATACTCGGATCAT	1211–1227	
	SCSK	F	ATGATGAAGACTATTTCTCCGGTGAG	1169–1191	
		R	GTCGGCCTCCATTCATCATG	1246–1226	
		P	CGGAGGCAGTCTATAC	1202–1219	

**Table 5:** Real-time PCR-assays for the detection of lyssavirus species other than RABV.

Genotype	PCR	Primer/Probe name	Role	Sequence	Position	Author
EBLV-2	TaqMan	JW12	F	ATGTAACACCYCTACAATG	55–73	[31]
		N165–146	R	GCAGGGTAYTTRTACTCATA	165–146	
		LysGT6	P	ACARAATTGTCTTCAARGTCCATAATCAG	81–109	
EBLV-1	TaqMan	JW12	F	ATGTAACACCYCTACAATG	55–73	[79]
		N165–146	R	GCAGGGTAYTTRTACTCATA	165–146	
		LysGT5	P	AACARGGTTGTTTTYAAGGTCCATAA	80–105	
		gt5L	F	GATCCCGATTTGAAAACAGC		
		gt5P	R	AGACCATGGCTCCAGCTAAA		
		AWgt5	P	GGGATGAATGCTGCTAAATTAGACCCA		
ABLV	TaqMan	LYSF-YB	F	GAACGCCGCGAAGTTGG	191–207	[70]
		LYSR-YB	R	AGATCCCCTCAAATAACTCCATAGC	240–264	
		LYSF-YB-FAM	P	CGGACGATGTTTGCTCCTACCTAGCTGC	211–238	
		LYSF-FF	F	TCGGGAATGAATGCTGCAA	183–201	
		LYSR-FF	R	GGCAGAYCCCCTCAAATAACTC	267–247	
		LYSF-FF-FAM	P	ACCCCGATGATGTATGTTCTTACTTAGCTGCAG	208–239	
ARV	TaqMan		F	CTTCGTCAAGGTGGTTGAACATC	531–553	[28]
			R	TGGAGCACGATACCAAATTTCA	589–610	
			P	CATTGATGACCACTCACAAGATGTGTGCAA	557–586	
KHUV	TaqMan		F	AACTGGGCATTGACCGGAG	355–373	[28]
			R	ACATGCATCCTTAGTGGGGCT	408–428	
			P	CTAGACCTGACAAGAGATCCGACTGTAGCCG	376–406	
IRKV	TaqMan		F	GTAATTGGGCTCAGGCAGGAG	353–373	[28]
			R	AGGAGCCCGACTAAAGACGC	412–431	
			P	ACAAGACCTTACTAGAGATCCAACAACACCGGAAC	375–409	

### Nucleic acid sequence-based amplification (NASBA)

The use of a rapid automated NASBA technique was successfully applied to the ante-mortem saliva and cerebrospinal fluid (CSF) of four rabies patients in Thailand and shown to have a ten-fold increase in sensitivity compared to RT-PCR [33]. The assay detected rabies viral RNA as early as two days after onset of symptoms. The NASBA technique involves the use of three enzymes (reverse transcriptase, RNase H and T7 RNA polymerase) to synthesise multiple copies of target RNA under isothermal conditions. Briefly, a large number of RNA copies are generated using a pair of specific primers, one of which contains the T7 RNA polymerase binding site, and the other which has an electrochemiluminescence detection region attached to the 5′ end. The amplified RNA is detected using an automated reader, enabling rapid throughput testing. It is relatively easy to use and the whole process from extraction to detection can take as little as four hours. This technology has already been applied for point of care testing of bacterial pathogens [34] and viral pathogens [35],[36]. The NASBA technique has also been adapted to investigate rabies virus replication *in situ*, whereby the relatively lower isothermal temperatures of NASBA compared to in-situ RT-PCR ensure that cell integrity is maintained [37].

### Loop-Mediated Isothermal Amplification (LAMP)

**Figure 1:**
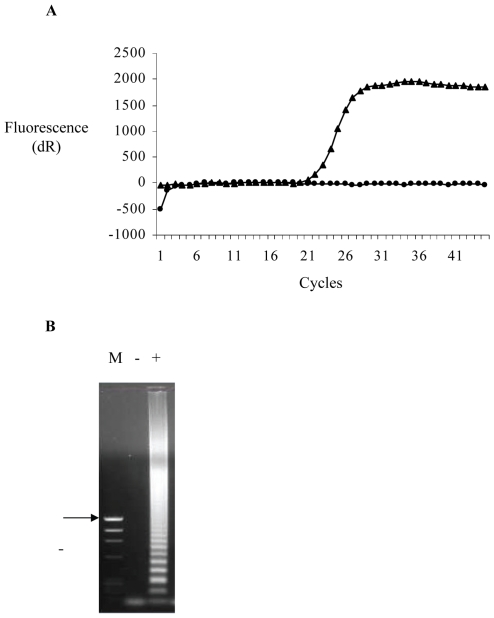
Amplification of rabies virus RNA by reverse transcription loop-mediated isothermal amplification (RT-LAMP). For each reaction 1 µg RNA, prepared using TriZol (Invitrogen) from normal mouse brain (1A, circles; 1B −) or infected mouse brain (1A, triangles; 1B, +) was added to a reaction mixture containing all six primers at concentrations indicated in Table 1, Thermopol buffer (New England Biolabs, USA), 0.2 mM dNTPs (Promega, UK), 2 mM MgSO4, 1 M betaine (Sigma, UK), 16 units Bst 1 polymerase (New England Biolabs), 0.12 units Thermoscript reverse transcriptase, 50 nM ROX dye (Invitrogen) and 1/1000 dilution of the intercolating dye picogreen (Molecular Probes) in a final volume of 25 µl. The reaction was incubated at 65°C for 1 hour in an MX3000P thermal cycler with data collection at 80 second intervals. Samples were analysed in real time (Figure 1A) or by separation in a 1% agarose gel (Figure 1B), the arrow indicates a marker band with a size of 1.35 kilobase pairs.

LAMP offers an alternative DNA amplification method to the polymerase chain reaction for applications to the ante-mortem saliva and CSF testing. The originators of the technique suggest that it amplifies with high specificity, efficiency and without the need for thermal cycling [38]. Amplification is achieved through the specific binding of two inner and two outer primers to the target sequence. The inner primers initiate strand synthesis whilst the outer primers displace the inner primers, allowing them to self-anneal to the nascent strand. This creates hairpin structures that trigger further strand synthesis that in turn lead to concatenation of the target sequence [38]. Polymerisation and strand displacement are achieved using a single enzyme, *Bst* 1 DNA polymerase. The technique is rapid, generating large quantities of target sequence within minutes. For the amplification of RNA viruses, a reverse transcription step is undertaken prior to the LAMP reaction. Primer sets have been successfully developed to detect a range of pathogenic viruses including West Nile virus [39], Japanese Encephalitis virus [40], Foot and Mouth Disease virus [41] and Chikungunya virus [42]. To assess the applicability of LAMP to the detection of rabies virus we designed a primer set using PrimerExplorer V4 software (Eiken Chemical Company Ltd., Japan) that can detect the Challenge Virus Standard (CVS) fixed strain of rabies virus (Table 6). In addition to the standard set of four primers, two further loop-binding primers have been added to increase the rate of strand displacement and synthesis [43]. The reverse transcription and LAMP reactions were undertaken simultaneously (RT-LAMP) in a single tube at 65°C using a thermostable reverse transcriptase, hence avoiding the step process inherent in an RT-PCR. Target amplification was monitored by the incorporation of the double stranded DNA binding fluorophore picogreen (Figure 1a) or by separation on a 1% agarose gel (Figure 1b). At a constant temperature of 65°C, CVS RNA could be detected within 30 minutes.

**Table 6:** Details of oligonucleotide primers designed to specifically amplify the Challenge Virus Standard strain of RABV.

Primer	Function	Sequence (5′- 3′)	Tm°C	Final Concentration
CVSF3	Forward outer primer	AGCCCCCGACTTGAACAAAG	67.3	5 picomoles
CVSB3	Backward outer primer	CTGTCAGAGCCCAATTCCCG	69.0	5 picomoles
CVSFIP	Forward inner primer	GCATTGCTGCTGCCAAGTAGGATTTTCAGGCATGAATGCCGCCAA	89.1	50 picomoles
CVSBIP	Backward inner primer	CATGTCCGGAAGACTGGACCAGTTTTATCTCCACTAGAGAGTTTGG	81.4	50 picomoles
CVSFLOOP	Forward loop-binding primer	GCATACATCCGGATCAAGT	64.7	25 picomoles
CVSBLOOP	Backward loop-binding primer	CTATGGAATCCTGATTGCACG	64.2	25 picomoles

Development of RT-LAMP assays for use in diagnosis and surveillance is challenged by the considerable sequence variation observed within the rabies virus genome [44] that can frustrate specific primer design. Preliminary attempts at this suggest that multiple combinations of primers (up to 12 different primers) can lead to sensitive, rapid amplification of RABV genomes from a wide range of geographical locations. The use of isothermal amplification has the benefit of reducing the technological requirements of thermal cycling used in RT-PCR. This in turn offers the opportunity, when linked with lateral flow devices, to develop surveillance protocols where testing can take place in the field or in less sophisticated laboratories.

### Microarray detection of lyssaviruses

**Figure 2:**
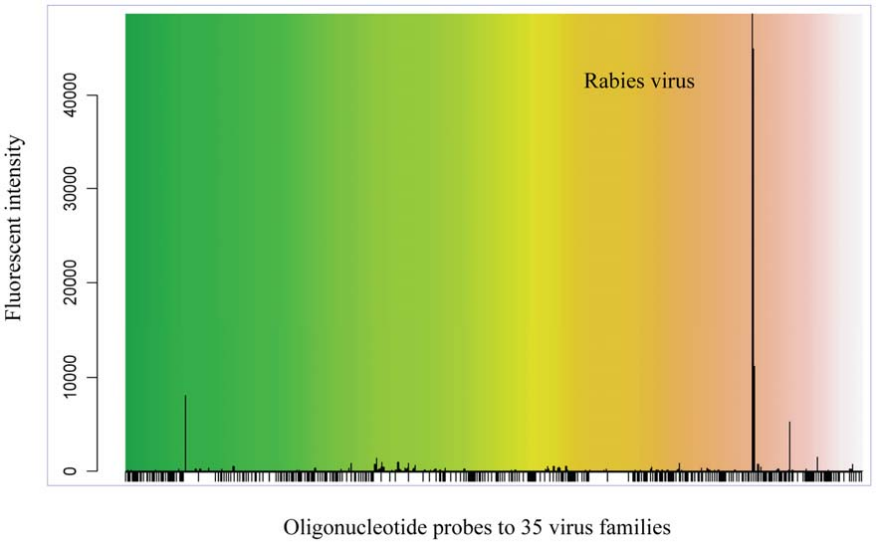
Microarray identification of rabies virus RNA prepared from a brain sample from a confirmed case of human rabies. Total RNA was extracted using TriZol (Invitrogen) and treated with DNase I prior to conversion to double stranded DNA [45]. Non-specific amplification was achieved using a DNA polymerase (Klen Taq, Sigma) and the products were labelled through binding of Alexa Fluor 555 reactive dye (Invitrogen) to amplicons. Labelled target DNA was denatured at 95°C and chilled on ice before dilution in hybridization buffer and addition to the microarray slide. Hybridization occurred at 50°C overnight. Slides were washed and the target-probe binding was captured using GenePix Pro 6.1 software (Molecular Devices). Statistical analysis of the data was conducted using DetectiV software [64].

Microarray linked to sequence independent PCR amplification offers the ability to rapidly identify pathogenic viruses from post-mortem samples [45],[46],[47]. We have undertaken a study that has demonstrated the ability of a microarray to detect each of the seven lyssavirus genotypes (VLA Weybridge, unpublished data). The microarray is composed of oligonucleotide probes 70 nucleotides in length and includes probe sets for each of the seven classified genotypes and sets for the unclassified lyssaviruses. The diagnostic capability of the array was illustrated showing the ability of the array to detect RABV in a human case of rabies as the amplified RNA bound specifically to the classical rabies virus (genotype 1) probe set (Figure 2).

## Antibody detection–based assays

### Development of a novel ultrasensitive and stable potentiometric immunosensor

A stable potentiometric immunosensor for the detection of various analytes of interest from complex real world samples such as blood, serum and milk has been described [48]. The biosensor detects enzyme labelled immunocomplexes formed at the surface of polypyrrole coated, screen-printed gold or silver electrodes. Detection is through a secondary reaction that produces charged products with a shift in potential, being measured by local changes in redox state, pH and/or ionic strength. The magnitude of the change in potential is directly related to the level of target in the matrix such that the assays are quantitative and the numerical output is rapidly transmissible. The bioassays produce very rapid results (5–15 minutes), are highly reproducible (%CV<5%) and are ultra-sensitive (<50 fM). Thus this sort of biosensor offers the rapidity of production of signal of a lateral flow device with the sensitivity of third generation ELISAs. Immunoassays can be developed in a sandwich or competitive format for small (e.g. Digoxin; MW 780 Da) or large (e.g. hepatitis B surface antigen; >300 kDa) molecules [49]. In addition to immunoassays, it is also possible to detect specific nucleic acids and whole cells using the technology. Due to the method of production of the electrode strips, the base assays are inexpensive (<US▒1 per strip) as is the detection hardware (US▒2,000). In addition, most immunoassays can be readily adapted to this format with minimal additional optimisation. Potentiometric immunosensors for detecting the rabies virus nucleoprotein are in progress and offer the ability to rapidly screen complex non-clarified matrices, possibly at pen-side, in a cost-effective manner.

### Development of lentiviral pseudotypes for the measurement or rabies virus neutralising antibodies

Serological assays are not suitable as diagnostic tools for routine rabies testing as the host response to infection varies substantially between individuals. However, serology is still useful, particularly to monitor the development of the immune response. We would suggest that detection of rabies antibodies in serum and CSF, early after presentation and in the absence of a history of vaccination may be a positive indicator for a therapeutic intervention. Viral pseudotypes, the core of one virus coated with envelope protein derived from a second virus, offers a safe alternative to the use of pathogenic viruses in neutralisation assays. Using pseudotypes expressing genotype 1 CVS-11 glycoprotein, high titre stocks (1.3–3.2×10^5^ infectious units/ml) were produced that proved 100% specific and highly sensitive compared with neutralisation titres achieved using the FAVN [50]. A high correlation was also observed (*r* = 0.89). Using pseudotypes expressing EBLV-1 (genotype 5) and EBLV-2 (genotype 6) G-proteins, neutralising antibody titres broadly correlated with the degree of G-protein diversity. A vaccine study in Tanzania compared the two assays with pseudotypes showing 100% specificity and 94.4% sensitivity to the FAVN with a high correlation of antibody titres (*r* = 0.92). Incorporation of Lagos bat virus (genotype 2), Mokola virus (genotype 3) and Duvenhage virus (genotype 4) G-proteins, as well as *lacZ* as a reporter gene, makes the pseudotype assay an attractive option for serosurveillance in Africa and other resource limited countries. In addition, as the pseudotype assay uses substantially less input serum (10 µl) compared to FAVN and RFFIT, multiple tests can be undertaken on samples where collection volumes are limited or valuable e.g. bat sera.

Due to the neurotropic nature of rabies virus, infection results in enormous viral replication in the CNS in the final stage of the disease that leads to massive antigen and viral genome concentrations. This makes detection of viral antigen in brain tissue by tests such as the FAT or the dRIT [7] very robust and relatively simple to perform, and these have become rapid gold standard tests. As for detection of viral genome, approaches are now available which process multiple specimens from nucleic acid extraction through to genetic typing, with significantly reduced risks of contamination. In addition, the use of TaqMan RT-PCR or similar technologies on robotics platforms, allow for rapid large-scale rabies detection, typing and quantification in real time [32],[31],[51]. The development of PCR-based methods (Box 2) provided an alternative method for post mortem rabies diagnosis [26], and the possibility of *ante mortem* diagnosis of human rabies [52]. RT-PCR methods invariably involve multiple transfers of nucleic acids between different tubes. Coupled with the high sensitivity of PCR methodologies, any small amount of contamination will undoubtedly produce false-positive results. Attempts have been made to adapt RT-PCR to reduce manipulations thereby reducing contamination risks. The visualisation of PCR products by gel electrophoresis exposes facilities and operators to large quantities of amplified material and thus many adaptations have been directed at replacing this step. New and improved rapid diagnostic tools for rabies using Taqman technology have been developed that avoid cross-contamination due to the closed tube nature of the test [29],[30]. A further benefit of RT-PCR has been to enable practical molecular characterization of rabies viruses [26] that has added significantly to the understanding of virus evolution and epidemiology. This approach has superseded the use of monoclonal antibodies for typing and characterising new strains of rabies virus. This has provided the evidence to support the delineation of lyssaviruses into genotypes [53] (Box 2) and was used for the classification of another four putative members of the genus [54],[55]. Also, this technique was a prerequisite for the understanding of the molecular biology of lyssaviruses [56] and underpinned future developments in rabies diagnosis and prevention. It is likely that in the future microarray techniques in combination with sophisticated bioinformatics and arrays with a hierarchical set of probes will provide an alternative to rapid virus discovery and characterisation.

The development of ‘real-time’ RT-PCR techniques allows the quantification of this RNA in ‘real-time,’ giving a relatively quick and reliable method for the measuring levels of viral RNA. PCR based techniques are not currently recommended by the WHO for routine post-mortem diagnosis of rabies. However, in laboratories with strict quality control procedures in place and demonstrable experience and expertise, these molecular techniques have been successfully applied for confirmatory diagnosis and epidemiological surveys. For these reasons, it is likely that international bodies will accept their use in the future for routine rabies diagnosis. Reverse transcription PCR has been reported to confirm rabies diagnosis *intra-vitam* in suspect human cases, when conventional diagnostic methods have failed and post-mortem material is not available (Box 1) [57]. Rabies virus RNA can be detected in a range of biological fluids and samples (e.g. saliva, CSF, tears, skin biopsy sample and urine). Owing to the intermittent shedding of virus, serial samples of fluids such as saliva and urine should be tested but negative results should not be used to exclude a diagnosis of rabies. All positive PCR results should be sequenced to confirm the origin of the virus and rule out possible contamination. In terms of the RNA concentrations in the brain, the sensitivity especially of nested or real-time PCRs may be beyond the threshold needed for routine post-mortem testing. Also, contamination of negative samples could lead to an unjustifiable administration of a high number of costly post exposure prophylaxis and would produce false data for the rabies surveillance. However, with the introduction of accreditation for laboratories, quality control measures are being implemented in a growing number of laboratories worldwide. Such quality controls for diagnostic rabies PCRs should encompass several measures, including the inclusion of appropriate positive, negative, and inhibition controls in assay runs. The consistency and the inter-assay reproducibility should also be ensured over time by monitoring performance. Only if laboratories meet the required standard [58], can PCR fulfil its full potential. The use of PCR should not be restricted only as a confirmatory diagnostic test for decomposed samples but also as a powerful tool for virus typing and molecular epidemiology studies. The lack of standardization is a major obstacle to the general use of PCR for rabies diagnosis, especially in developing countries.

It is evident that the RT-PCR dominates genetic detection of rabies virus and it seems probable that this technique will dominate rabies diagnosis in the 21^st^ century. However, we should not discount alternatives that have the benefit of isothermal amplification that will enable implementation in laboratories where access to thermal cyclers is an obstacle. A NASBA technique was successfully applied to the saliva and CSF of four living patients with rabies and detected rabies viral RNA 2-days after the onset of symptoms. This technique has also been adapted to investigate rabies virus replication *in-situ*. LAMP also falls into this category and can be adapted for use with lateral flow devices thus making its application very simple.

Existing assays for rabies virus antibody prevalence studies either require high containment facilities or do not distinguish between neutralising and non-neutralising antibodies [59]–[60]. Recently however, a neutralisation assay using retroviral pseudotypes was described [50], not bound by the restrictions listed above and also allowing a choice of endpoint reporter proteins (β-galactosidase, green fluorescent protein or luciferase) [61]. A further benefit of this technique is its adaption to using small volumes of sera thus making them useful for surveillance.

Currently, high-throughput rabies virus molecular detection methods augment standard diagnostic tests or are in the process of development and refinement for use alone. As we progress through the 21^st^ century, it is possible that these techniques will replace conventional tests (Box 1). Obstacles to adoption include cost, complexity and local acceptance of their use. It is also possible that immunological tests by measuring ‘indirect’ markers such as cytokines and electrolytes will increase in use. These tests however, will probably remain in the realm of large reference laboratories where resources allow the development of novel assays. As far as semi-automated or automated instruments and robotics-based techniques are concerned, they are useful when large numbers of the same test are undertaken such as surveillance and companion animal testing and these tests will continue to increase in popularity and use, especially in central reference facilities. There is a clear need to simplify molecular diagnostic techniques so these tests can be applied universally in developing and developed countries. It is likely that new developments will focus on generating low volume and yet affordable diagnostic tests for rabies. More use will be made of point-of-care (POC) diagnostic testing using portable extraction techniques linked to PCR machines with the use of lyophilised reagents to overcome cold-chain dependencies in tropical countries. In the 21^st^ century, these technologies will have a demonstrable impact on people living in developing countries, especially where rabies is still considered a ‘neglected’ disease. By contrast in the developed world, these new technological advances will undoubtedly be faster, more accurate and cost-effective leading to a ‘Theragnostics Approach’ that combines therapeutics with diagnostics for the human treatment of rabies. Interest in treating human rabies aggressively is gaining momentum, largely due to the reported success in treating a 15-year-old girl, in whom clinical rabies developed one month after she was bitten by a bat, using a combination of therapeutic coma with antiviral drugs whilst allowing for the host immune system to confer immunity – The ‘Milwaukee Protocol’ (Box 2) [62],[63]. Bioinformatics, genomics, proteomics, and functional genomics are the molecular biology tools that are essential for the progress of molecular ‘theragnostics’, where both early diagnosis and monitoring of serology are critical factors for the successful treatment of a rabies patient. In addition, theragnostics could eliminate the unnecessary treatment of patients for whom rabies immunotherapy is not appropriate i.e. immunosuppressed patients, resulting in substantial drug cost savings for the healthcare system.
